# The Construct Validity of Intellect and Openness as Distinct Aspects of Personality through Differential Associations with Reaction Time

**DOI:** 10.3390/jintelligence11020030

**Published:** 2023-02-01

**Authors:** Emily A. Willoughby, Yuri Kim, James J. Lee, Colin G. DeYoung

**Affiliations:** Department of Psychology, University of Minnesota Twin Cities, Minneapolis, MN 55455, USA

**Keywords:** Big Five Aspect Scale, personality, reaction time, cognitive ability, *g*, additive factors

## Abstract

The construct validity of group factor models of personality, which are typically derived from factor analysis of questionnaire items, relies on the ability of each factor to predict meaningful and differentiated real-world outcomes. In a sample of 481 participants, we used the Big Five Aspect Scales (BFAS) personality questionnaire, two laboratory-measured reaction time (RT) tasks, and a short-form test of cognitive ability (ICAR-16) to test the hypothesis that the Intellect and Openness aspects of Big Five Openness to Experience differentially correlate with reaction time moments. We found that higher scores on the Intellect aspect significantly correlate with faster and less variable response times, while no such association is observed for the Openness aspect. Further, we found that this advantage lies solely in the decisional, but not perceptual, stage of information processing; no other Big Five aspect showed a similar pattern of results. In sum, these findings represent the largest and most comprehensive study to date on personality factors and reaction time, and the first to demonstrate a mechanistic validation of BFAS Intellect through a differential pattern of associations with RT and Big Five personality aspects.

## 1. Introduction

Reaction time (RT) as measured by elementary cognitive tasks reflects the speed of perception, decision, and motor response to a relatively simple stimulus. General cognitive ability (*g*) is perhaps the most well replicated predictor of individual mean RT, with nearly a century of data showing that individuals who score higher on cognitive ability tests tend to have faster, more accurate, and less variable reaction times (Deary et al. 2001; Hunt 2005; Jensen 1982; Sheppard and Vernon 2008). In a literature review by Sheppard and Vernon (2008), the mean correlation between various measures of mental speed and intelligence was −.24 (*SD* = .07).

Whether and to what extent there exists a relation between personality factors and reaction time has remained largely unexplored in personality literature, despite the inclusion of a cognitive-adjacent factor in most modern personality taxonomies, such as the Big Five model (Abe 2005; Digman 1990; Goldberg 1993; John 1990; John et al. 2008; McCrae and Costa 1997b; Wiggins 1996; Wiggins and Trapnell 1997). This is perhaps most surprising for the domain of Openness/Intellect, which has repeatedly emerged as the only Big Five factor that correlates significantly (r≈.30) with measures of cognitive ability (Ackerman and Heggestad 1997; DeYoung 2020; DeYoung et al. 2014; Schwaba 2019). A recent large-scale (*N* > 162 thousand) meta-analysis found Openness/Intellect to be the only domain that was a significant positive predictor of intelligence (ρ = .20; Anglim et al. 2022). This domain reflects variability in the tendency to seek and appreciate artistic and intellectual pursuits, and has been labeled in the literature over time first as Culture (Norman 1963; Tupes and Christal 1958), then as Intellect (Goldberg 1990; John 1990), and finally as Openness to Experience, reflecting the finding that measures of intellectual interest co-varied with measures of aesthetic sensitivity (McCrae and Costa 1997a). However, other researchers have argued that neither Openness to Experience nor Intellect alone sufficiently describes the broadness of the fifth domain, suggesting instead that the domain is composed of two subcomponents that directly correspond to Openness and Intellect (DeYoung et al. 2007; Jang et al. 2002; Woo et al. 2013). Some authors have opted for the compound label of Openness/Intellect (Oleynick et al. 2017), which we use throughout this manuscript.

More recently, researchers have proposed that two distinct but correlated factors underlie each Big Five dimension. The instrument used to measure these factors has been formalized as the Big Five Aspect Scale (BFAS; DeYoung et al. 2007), and it is able to subdivide variance within the Openness/Intellect factor into Intellect—an aspect that represents variation in engagement with semantic information and sensitivity to logical or causal patterns—and Openness, which captures sensitivity to patterns in perceptual information over time and space (DeYoung 2015; DeYoung et al. 2010). Indeed, the correlation of Openness/Intellect with general intelligence appears to be driven entirely by Intellect, as the Openness aspect is no longer correlated with *g* after controlling for Intellect (DeYoung et al. 2014). One recent study, which found that the trait Intellect uniquely predicts the allocation of cognitive resources between two working-memory tasks, suggests that one mechanistic function of Intellect is to prioritize cognitive resources in attending to a primary task (Smillie et al. 2016). More of such research is needed to demonstrate evidence that Intellect is a distinct dimension of personality that can make unique behavioral predictions that go beyond a questionnaire self-report.

Given the compelling view of cognitive ability as a component of personality (e.g., DeYoung 2020), it is perhaps surprising that few studies have been conducted to date of reaction time and Big Five personality dimensions. Several of these have only reported relations between RT and Extraversion or Neuroticism (Gupta and Nicholson 1985; Stelmack et al. 1993). For example, Rammsayer et al. (2014) found in a sample of 63 high and 63 low Extraversion participants that higher levels of Extraversion were associated with faster responses, though the authors note this is likely a function of specific task demands rather than underlying cognitive differences, as Extraversion does not appear to correlate with *g* (DeYoung 2020). Others have noted a modest negative association (r<.20) between Neuroticism and mean RT in choice reaction time tasks, as well as somewhat larger associations (r≈.25) between Neuroticism and the variability of participant responses (Robinson and Tamir 2005).

However, much of this previous research tends to be limited by a number of issues, including small (N<100) and homogeneous (e.g., all female) samples and the use of personality questionnaires that do not distinguish between aspects of a given factor. Further, many of these studies report results for only those personality factors which yield significant associations, leading to uncertainty about whether these were the only factors studied or if the authors failed to report non-significant results, the latter of which poses a significant problem for replicability (Button et al. 2016). Openness/Intellect and its aspects are largely absent from previous literature investigating the relation of personality and RT, and the strong empirical and conceptual relation between these factors and cognitive ability makes the BFAS uniquely well suited for investigations of reaction time.

### 1.1. Construct Validity of Personality Taxonomies

Fundamental to the validity of psychological constructs is their ability to meaningfully and differentially predict behavioral outcomes (Cronbach and Meehl 1955; Loevinger 1957; Messick 1989). Reaction-time moments are widely used to infer some basic quality of information and sensory processing (Luce 1986), and may therefore represent a useful behavioral outcome to differentiate individual differences in lower-order personality factors. The present study investigates the association between personality aspects and reaction time with the Big Five Aspect Scale and two reaction-time tasks that differ in sensory modality (auditory and visual), thereby testing the hypothesis that performance on reaction time tasks is uniquely associated with some, but not other, aspects of personality.

One common criticism of psychological constructs derived by factor analysis of questionnaire items is that they rely on inadequately incisive method of distinguishing factors, leading some researchers to question their psychometric validity (e.g., Block 1995; Eysenck 1994). Much of these criticisms have questioned the ability of Big Five factors to differentially predict meaningful behavioral outcomes (McAdams 1992); others accept the validity of the Big Five but dispute a particular model of the lower-order factors. Competing models of *g* have undergone similar skepticism: For example, the validity of the 10 lower-order factors beneath *g* posited by John Carroll’s factor analytic output of IQ subscales (Carroll 2003) continues to be disputed.

A similar approach is to find for each factor a distinct causal or mechanistic basis. Such biological correlates might include genetic variants, brain systems, volumes of different brain regions, while elementary cognitive expressions may include reaction time on structured laboratory tasks. This prediction has borne out for individual differences in factors such as Neuroticism, Openness/Intellect, and Extraversion, which have been found to be differentially associated with distinct genetic variants (Nagel et al. 2018), fMRI activity supporting working memory and functional connectivity in dopamine-rich networks (DeYoung et al. 2009; Passamonti et al. 2015), and decreased latent inhibition (Peterson et al. 2002), respectively. Moreover, it has been proposed that different dopaminergic subsystems may underlie variation in Extraversion and Openness/Intellect by facilitating engagement with cues of reward and the salience of information, respectively (DeYoung 2013). These findings have been largely consistent with theories of personality which posit that individual variation in personality reflect underlying strategies for goal-attainment (DeYoung 2015; DeYoung et al. 2010), and they provide a promising foundation on which to test the hypothesis that performance on elementary cognitive tasks is a plausible mechanistic correlate of variation in some, but not other, dimensions of personality. By providing evidence for RT being related to Intellect but not Openness, the present study is contributing to such construct validation for the BFAS.

### 1.2. The Current Study

In this manuscript, we report results from a large sample (N≈500) of participants measured on two choice RT paradigms adapted from well known laboratory tasks of numerical and auditory discrimination (Moyer and Landauer 1967; Sigman and Dehaene 2005), a validated short-form assessment of cognitive ability (ICAR-16; Condon and Revelle 2014), and the Big Five Aspect Scale (BFAS; DeYoung et al. 2007). Effects are reported from correlations of RT moments with both the five factors and the ten aspects of personality computed from the BFAS.

In addition to exploring these correlational relationships, our two RT paradigms allow us to further separate perceptual and decisional stages of information processing in reaction time tasks. In a previous study, we applied Sternberg’s method of additive factors by systematically manipulating the demands on perceptual acuity and decision-making in order to test the hypothesis that general cognitive ability is associated with one and only one of these stages (Willoughby and Lee 2021). The present study uses the same reaction time paradigms to test a hypothesis suggested by DeYoung (2020)’s descriptions of the cybernetic functions of the Intellect and Openness aspects of Openness/Intellect. This theory suggests that the Intellect aspect should predict sensitivity to logical patterns, and that the Openness aspect predicts sensitivity to perceptual patterns. If Intellect and Openness represent sensitivity to patterns in semantic and perceptual information, respectively, then it is plausible to predict that each of these aspects would interact differentially with experimental manipulations of semantic and perceptual difficulty in a reaction time task.

## 2. Materials and Methods

As per the recommendation of Simmons et al. (2012), we report how we determined our sample size, all data exclusions, all manipulations, and all measures. Details are available in Appendix A.

This project was preregistered on the Open Science Framework (https://osf.io/q792f/?view_only=33e1fb84883045bb9be6a4d2dff35e55, accessed on 18 February 2021). One deviation from the preregistration is important to note (Nosek et al. 2019): While a mediation analysis was originally planned, the authors decided that mediation was inappropriate in this case, as it is inadequate in assessing causal paths.

### 2.1. Participants

Participants were recruited through the undergraduate psychology recruitment pool at the University of Minnesota. A total of 481 participants took part in the experiment over a 3-year period; of these, 477 had valid data for all four tasks (M=19.6, SD=1.6; 77.8% female). To account for well known ontogenic changes in reaction time (Thompson et al. 2014), individuals were recruited from the 18–24 age range. Participants were additionally required to be comfortable with English instructions to ensure full understanding of task requirements, and were required to have normal or normal-corrected hearing and vision. Each participant gave written consent before beginning the experiment, and experiments were approved by the University of Minnesota Institutional Review Board in accordance with the ethical principles of the Belmont Report.

This project was established as an extension of the project described in Willoughby and Lee (2021), and the present sample (*N* = 477) represents a subset of this previous sample (*N* = 773) of individuals who had provided reaction time and ICAR-16 data and were subsequently administered the Big Five Aspect Scale for the present study.

#### Power Analysis

To justify the target sample size of 500 individuals, we conducted power analyses at two levels of analysis. At the first level, 500 participants would permit power of at least 80% to detect a correlation as small as *r* = −.16 with a Type I error rate of .005, and as small as *r* = −.18 with a Type I error rate of .001. Although anticipating the power of a covariate in a 2 × 2 ANOVA relies on this correlation as well as the effect sizes of each manipulation, power analysis of 500 participants over 4 levels of one manipulation is able to detect an F-ratio as small as .09 with 80% power and a Type I error rate of .005. In reference to previous work using the same reaction-time tasks, the main effect of each manipulation in Willoughby and Lee (2021) ranged from *F* = 67.0 to *F* = 1200.0; these main effects are unlikely to be substantially different in an experiment using the same tasks.

### 2.2. Measures

#### 2.2.1. Big Five Aspect Scale

**Figure A1:**
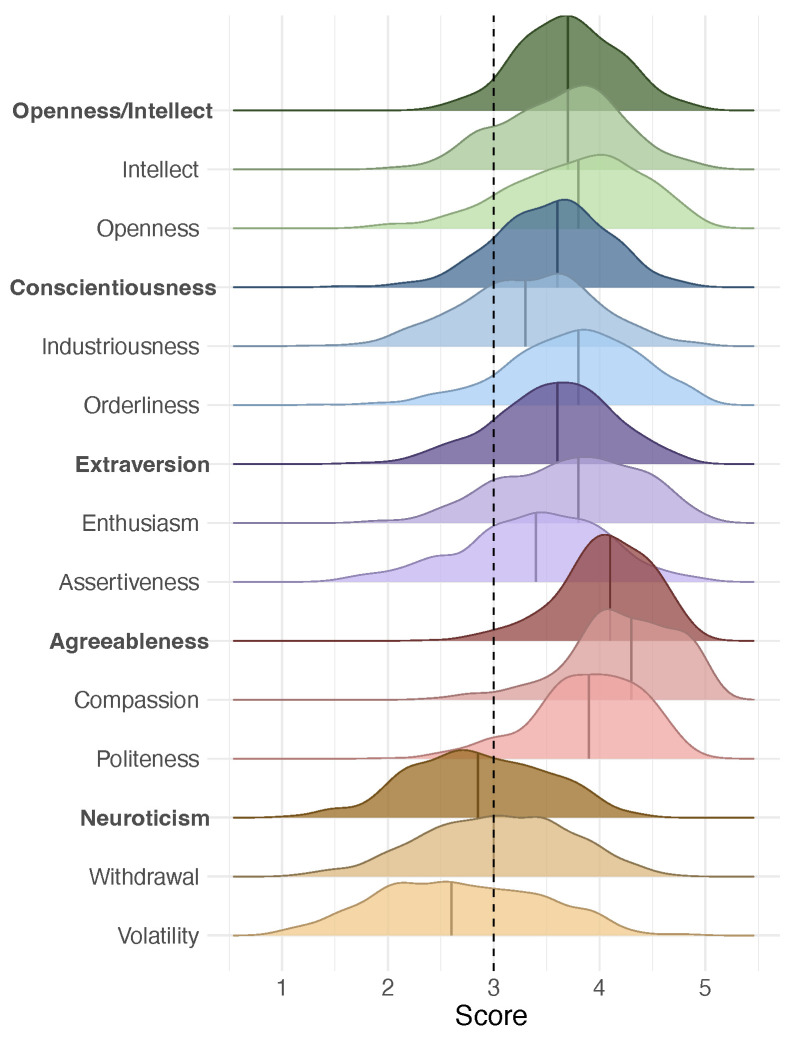
Joy plot showing distributions for each Big Five factor and aspect in the current sample (*N* = 480). Dashed line represents theoretical mean, and individual solid lines represent medians for each factor or aspect.

Big Five personality domains and their 10 aspects were assessed using the Big Five Aspect Scale (BFAS; DeYoung et al. 2007). Responses are given on a 5-point Likert scale, and each aspect is computed from 10 items. Big Five domain scores are then computed from the average of each pair of corresponding aspects. Descriptive statistics for BFAS scores in our sample are shown in Table 1. See Appendix B Figure A1 for sample distributions, and Appendix B.1 for model-fit analyses.

#### 2.2.2. ICAR-16 Sample Test

The cognitive test consisted of a short-form assessment chosen for its reliability, confirmed loading on *g*, and relative quickness. We used the ICAR-16, a 16-item, multiple-choice short form of the full public domain International Cognitive Ability Resource assessment (icar-project.com; Condon and Revelle 2014, accessed 31 January 2023). Participants were not given a strict time limit, and all completed it within 30 min. The sample test consists of 16 items taken from the full 60-item ICAR test, each of which comprises one of four item types or subtests. These four subtests are summarized as letter and number sequences (LN), matrix reasoning (MR), 3D rotation (R3D) and verbal reasoning (VR). Note that ICAR-16 reliability is moderately lower than the full 60-item ICAR; lower internal consistency is likely due to the lower number of items in the sample test (see, e.g., Ziegler et al. 2014a, 2014b). Reliability statistics and comparisons with Condon and Revelle (2014)’s validation sample are shown in Table 2, and additional item analysis can be found in Appendix B.1 (see Table A2 and Table A1).

#### 2.2.3. Reaction Time Paradigms

Participants’ reaction times were generally slower, more variable, and had higher error rates in the tone comparison than in the number comparison task. Sample mean, standard deviation, range and skew are shown in Table 3 for the ICAR-16 and individual means, standard deviations and accuracy of reaction time on both tasks. Mean reaction times on both tasks were positively skewed, as is typical on response time tasks.

### 2.3. Design and Procedure

In addition to capturing RT moments across two tasks of differing sensory modality, the reaction time tasks were designed to employ additive-factors logic in order to compare the effects of two manipulations on task performance. Both manipulations were intended to slow down performance, but only one was intended to achieve this slowdown by varying the difficulty of discriminating the trial stimulus from other members of the stimulus set. The second manipulation, by contrast, varied the difficulty of perceiving the stimulus.

The first experimental paradigm focuses on visual processing of numerals displayed on a computer screen. We chose a number-comparison task from the field of numerical cognition, where detailed mechanistic models of robust phenomena have already been proposed (Cohen Kadosh and Dowler 2015; Dehaene 2007). In such a task, participants are given a target digit that is invariant across all trials. In any particular trial, they must indicate via keypress whether the stimulus digit is less than or greater than the target. For the perceptual manipulation, we varied the contrast of the numerals against the background across four levels; lower contrast produces slower responses. This manipulation was intended to target an early stage of visual processing that should occur temporally prior to any processing of informational content. That this manipulation would target an early stage of visual processing is supported by many lines of converging evidence (Campbell et al. 1973; De Valois et al. 1982; Hubel and Wiesel 1968; Sigman and Dehaene 2005). The stimulus digits (1 through 9 excluding 5) were randomized across trials. Additionally, the four levels of Contrast were also randomized across trials to produce a total of 16 Distance × Contrast combinations. Participants first completed 30 practice trials, then 3 blocks of 50 trials, each of which were separated by short breaks. A given combination of digit and level of Contrast could only appear a maximum of three trials in a row in order to mitigate the unwanted influence of stimulus repetition (e.g., Kraut and Smothergill 1978).

The tone comparison task is designed to be structured analogously to the number-comparison task. Following a randomized foreperiod delay between 1200 and 1900 ms, two tones are presented sequentially to the participant through the set of headphones connected to the computer. The first tone is the target, which is positioned at the center of the distribution of stimulus frequencies (660 Hz) and at normal speaking volume (50 dB). The second tone is the stimulus, which can be any combination of frequency and loudness from the set of 16 options, excepting the target frequency of 660 Hz. The participant is instructed to respond as quickly and accurately as possible by pressing the “Q” key on the keyboard if the second tone is higher in pitch than the target tone, and the “W” key if the stimulus tone is lower in pitch. A keypress terminates the trial and initiates brief feedback (correct or incorrect) before continuing to the next trial. Eight total stimulus frequencies were used, four above and four below the target frequency; in this condition “distance” is coded as distance from the target frequency for four levels of “distance” and a total of 16 different conditions per participant. The four levels of both loudness and frequency distance are randomized and counterbalanced across trials. Each stimulus frequency and loudness level can appear a maximum of three trials in a row. Participants first complete 30 practice trials, then 3 blocks of 50 trials each which are separated by short breaks.

### 2.4. Analysis

All data were analyzed in *R* R Core Team (2013). ANCOVA was conducted using the *ezANOVA* function of the EZ (v4.4-0) package, and confidence intervals testing for difference between dependent correlations with *R*’s *cocor* package (Diedenhofen and Musch 2015).

Trials were dropped if their times were below 100 ms or more than 5 SDs from that participant’s mean RT. Trials resulting in an incorrect response were also dropped in analyses of raw RT. Participants with too few entries per condition were dropped from the final analysis (see Appendix A.2 for details of data-exclusion criteria).

The method of additive factors depends on finding statistical interactions between experimental manipulations or naturally varying traits associated with a common information-processing stage (and, conversely, an absence of interactions between manipulations or traits associated with distinct stages). We tested for the presence or absence of these interactions following Willoughby and Lee (2021); instead of using ICAR-16 score as a continuous between-subjects covariate in ANCOVA, we test for interaction effects with Big Five aspects, particularly Intellect and Openness, as the covariate of interest, thereby testing the hypothesis that Intellect, for example, confers a similar advantage across levels of decisional difficulty as we previously found for ICAR-16 score.

We recognize the perils of interpreting marginally significant *p*-values in light of multiple testing in presenting exploratory associations between all 5 domains and 10 BFAS aspects of personality with various RT moments. Due to the incompleteness in the existing literature of documented associations (or lack thereof) of RT with dimensions of personality, we have opted to report these effects in their entirety. As per the recommendation of Benjamin and Berger (2019), we use an alpha level of .005 for statements of significance; *p*-values between .005 and .05 are referred to as “nominally significant” or “suggestive”. Nevertheless, the reader is advised to interpret relationships of nominal significance through a lens of existing theoretical justification.

## 3. Results

Big Five domains and aspects intercorrelated as expected, with each aspect correlating with its domain in the r≈ .80 to .90 range (all p<.001). Correlations between aspects of a given factor were more variable, ranging from *r* = .24 for Openness with Intellect (aspects of Openness/Intellect) up to *r* = .58 for Withdrawal and Volatility (aspects of Neuroticism). All correlations between aspects within a factor were significant at p<.005. ICAR-16 score correlated with the Openness/Intellect domain at *r* = .13 (*p* = .004) and with the Intellect aspect at *r* = .24 (p<.001).

Additionally, we partially replicate findings from DeYoung et al. (2014) in the differential pattern of correlations between *g*, verbal and nonverbal intelligence, and aspects of Openness/Intellect (Table 4). Although Intellect correlates reliably with all subtest measures of the ICAR-16, Openness fails to correlate with verbal reasoning as expected, though this is likely due to the insufficiency of the four verbal reasoning items in capturing verbal intelligence.

Table 5 shows all interrelationships between BFAS measures, reaction time moments, and the ICAR-16.

### 3.1. Relationships between Personality and RT Moments

As expected, the short-form measure of cognitive ability correlated significantly with mean RT on both number (*r* = −.38) and tone (*r* = −.27) tasks, and additionally correlated with RT *SD* on both tasks (*r* = −.29 and *r* = −.23 for number and tone tasks, respectively) and accuracy only on the tone task (*r* = .27). This pattern of correlations (all p<.001) mirrors those from the same tasks in Willoughby and Lee (2021) of which the current study is a subsample, indicating that individuals with higher cognitive ability scores are faster, less variable, and more accurate (in the case of the tone task) in their responses.

#### 3.1.1. RT Moments and Big Five Domains

The domain of Openness/Intellect emerged as the only Big Five factor that was associated significantly or nominally with mean reaction times on either task (Table 5). For the number task, Openness/Intellect correlated with mean RT at *r* = −.13 (*p* = .005); for the tone task, this relationship was *r* = −.16 (p<.001). Response variability was also correlated at nominal significance with Openness/Intellect at *r* = −.11 (*p* = .02) for number RT *SD* and significantly at *r* = −.15 (*p* = .001) for tone RT *SD*. Accuracy was significantly correlated with Openness/Intellect in the tone task (*r* = .13, *p* = .004). Additionally, Conscientiousness was found to correlate with nominal significance with accuracy on the number task only (*r* = .12, *p* = .008).

#### 3.1.2. RT Moments and Big Five Aspects

When Big Five domains are separated into pairs of aspects, a clearer picture of the true nature of their relationship with RT moments emerges (Table 5). While the Openness aspect of Openness/Intellect failed to correlate suggestively with any RT moment, the Intellect aspect showed a clear pattern of intercorrelation with mean RT on both number (*r* = −.18, p<.001) and tone tasks (*r* = −.21, p<.001). Standard deviation of RT response showed a similar pattern of relationships with Intellect on both tasks, though accuracy correlated with Intellect significantly only for the tone task. Altogether, this pattern of results is suggestive of the Intellect aspect as driving the more modest Openness/Intellect correlations with RT moments, with individuals scoring higher on Intellect generally having faster, less variable, and more accurate RT responses.

Although no other BFAS aspect correlated at p<.001 with mean RT or variability on either RT task, it is worth noting several small but significant correlations with RT accuracy. For accuracy on the number task, Orderliness (*r* = .13, *p* = .0043) and Agreeableness (*r* = .13, *p* = .0049) both correlated significantly, with Conscientiousness reaching nominal significance (*r* = .12, *p* = .008). For accuracy on the tone task, Compassion correlated significantly (*r* = .13, *p* = .003) and Agreeableness nominally (*r* = .12 *p* = .008). Though these effects are small, the patterns of association make some theoretical sense: Participants with a greater tendency towards order may be motivated to avoid errors, as error detection rate has been found to be correlated with Conscientiousness in other types of task (Schell and Reilley 2004). Accuracy and Agreeableness may be related in that more agreeable people are thought to care more about cooperation and goal coordination (DeYoung 2015), and therefore may be more likely to follow instructions such as “Please try to be as accurate as possible.” Why the Compassion aspect (but not Politeness) is associated with accuracy on both tasks ($r^{2}$ = .014–.018) is harder to explain on theoretical grounds, and the association fails to attenuate when controlling for Intellect and ICAR-16, with a partial (Cohen’s) $f^{2}$ for Compassion of .017 for accuracy on the tone task and $f^{2}$ = .018 for accuracy on the number task. However, these modest correlations fail to achieve significance when a Bonferroni correction for multiple comparisons is applied (Appendix B Table A3).

### 3.2. Does IQ Account for the Intellect–RT Relationship?

For clarity of inference, we tested for a significant difference between correlations of Intellect and Openness with IQ and RT moments with Zou (2007)’s method for computing confidence intervals for the difference between two dependent correlations. As expected, the Intellect aspect had a significantly stronger correlation with IQ than the Openness aspect, while among RT moments the Intellect aspect had a significantly stronger correlation with various RT moments on both tasks; however, these associations were attenuated or eliminated at the more stringent CI of 99.5% CI (Table 6).

Next, we investigated the question of whether cognitive ability attenuated or eliminated the effect of Intellect on mean RT through multiple regression. Although Intellect, IQ, and RT are all intercorrelated, Intellect and IQ appear to contribute *unique* sources of variance to the production of speeded responses. IQ scores in our sample explain approximately 14% and 7% of the variance in mean RT in the number and tone tasks, respectively. In the number task, multiple regression including both Intellect and ICAR-16 scores as predictors yields an increment added by Intellect of less than one percent (*p* = .03), while the same prediction for mean RT in the tone task reveals Intellect as contributing an additional 3% variance (p<.001). Put another way, the partial (Cohen’s) $f^{2}$ for Intellect in predicting mean number RT declines from .034 to .010 with the inclusion of ICAR-16 as a covariate; for the tone task, the partial $f^{2}$ of Intellect declines from .047 to .025 when IQ is included. In sum, this analysis indicates that both cognitive ability and the Intellect aspect contribute small but unique sources of variance to reaction time in the tone task, while RT in the number task is chiefly explained by cognitive ability. Implications and limitations of this analysis are considered in the Discussion.

### 3.3. Effects of Experimental Manipulations

Main effects of both manipulations were revealed through repeated-measures ANOVA. For the number comparison task, strong and significant main effects were found for Distance ($F_{3,1425}$ = 835.5, $\eta_{p}^{2}$ = .638) and Contrast ($F_{3,1425}$ = 368.1, $\eta_{p}^{2}$ = .437). The tone task yielded a similarly strong main effect of frequency Distance ($F_{3,1425}$ = 350.6, $\eta_{p}^{2}$ = .425), albeit there was weaker evidence of a main effect for tone loudness (Contrast) of $F_{3,1425}$ = 22.2 ($\eta_{p}^{2}$ = .045). All main effects of manipulations on mean RT were significant at p<.005. In other words, increasing levels of difficulty for both manipulations yielded reliable slow-downs of mean reaction time for both tone and number tasks. Means and standard deviations of RT and proportion correct across levels of difficulty are shown in Table 7.

#### Interactions with Aspects

Given the strong main effects of manipulations in both tasks, we can investigate whether personality aspects, particularly Openness and Intellect, statistically interact with one, both, or neither manipulations. Evidence of such a statistical interaction with one of these manipulations would indicate that the given aspect confers a benefit in RT that scales along with the difficulty of the manipulation. For example, Willoughby and Lee (2021) found in the same RT paradigms that cognitive ability shows such an interaction with Distance, but not Contrast, indicating that general intelligence facilitates less of a slow-down at higher levels of difficulty for decisional, but not perceptual, stimulus content. It is plausible that Intellect or Openness are viable personality candidates for a similar effect.

We tested this hypothesis with Intellect and Openness as continuous covariates in both tone and number ANCOVA models. One participant, who was missing valid data for one cell mean, was removed for these analyses (*N* = 476). For the number comparison task, no significant evidence was found for an interaction of Distance with Intellect ($F_{3,1425}$ = 2.8, p=.05, $\eta_{p}^{2}$ = .006); it is unclear whether this is because our sample was underpowered to detect such an effect or if it arises from a more fundamental property of the expression of these aspect scores in the mental processing of numerical stimuli. For the tone comparison task, however, a significant effect was found for the interaction of Intellect with Distance ($F_{3,1425}$ = 7.2, *p* = .002, $\eta_{p}^{2}$ = .015), while no evidence was found for a simultaneous interaction with Contrast (*p* = .11). No evidence was found for a moderate or significant interaction of either manipulation with Openness. All *p*-values reported for covariate interactions represent Greenhouse–Geisser sphericity corrections.

**Figure 1:**
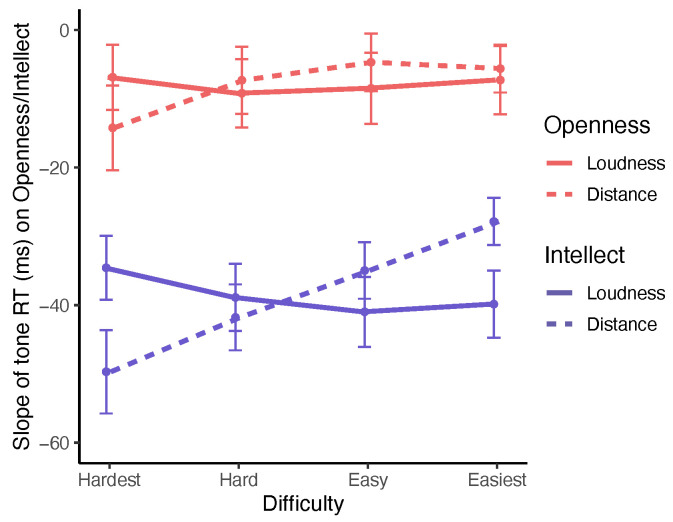
Regression coefficients of RT (ms) on the BFAS Intellect (bottom lines) and Openness aspects (top lines) across levels of difficulty for both tone loudness and frequency distance manipulations in the tone comparison task. Aspect scores represent standard scores. Error bars represent ±1 standard error of the sample mean.

To visualize the patterns suggested by this ANCOVA, we compared slopes across levels of difficulty for both manipulations that result from regressing RT means on aspect scores. If Intellect is acting as a significant moderator of Distance, we would expect to see the slope of this relationship change monotonically across difficulty levels in the simultaneous absence of such an effect over Contrast (loudness) levels. This pattern of results is shown in Figure 1, and strongly supports the conclusions from ANCOVA. The slope of RT on Intellect is −49.7 (SE = 6.1) in the hardest Distance condition and −27.8 (SE = 3.4) in the easiest condition; this monotonic change over Distance levels suggests that higher scores on Intellect facilitate the speeded processing of decisional information in the tone comparison task. The comparable pattern of slopes for Openness is in the same direction, but its failure to achieve significance in ANCOVA is represented by a much smaller difference between the slope of RT on Openness in the hardest condition (–12.5, SE = 6.1) versus the easiest condition (−4.2, SE = 4.4). No pattern for either aspect is evidenced by slope changes over levels of Contrast (tone loudness).

## 4. Discussion

Psychologists have long debated whether and to what extent intelligence should be integrated with models of personality on both theoretical and measurable grounds. The view that personality and intelligence represent distinct domains with only incidental intersection appears to be losing consensus, as recent empirical research continues to strengthen the prospect that such an integrated model can provide greater clarity in revealing true sources of individual differences (DeYoung 2020). Over the past two decades, empirical studies have consistently shown that intelligence is related to the Big Five domain of Openness/Intellect (DeYoung 2020; Stanek 2014) and especially to its Intellect aspect, which appears to capture meaningful variance in an individual’s perceived intelligence and intellectual curiosity (DeYoung et al. 2007). A cybernetic approach to personality taxonomy provides a compelling model wherein the mechanistic expression of Intellect and its relationship with intelligence can be empirically tested.

Our findings lend additional support to the perspective that this integrated model represents a natural property of personality variation. In a sample of nearly 500 participants, we examined the relation of Big Five aspects to a lower-level expression of individual variation that has largely been ignored in personality psychology: response times on elementary cognitive tasks. In both an auditory and a visual reaction time paradigm, we showed that average speed of response correlated significantly with the Big Five dimension of Openness/Intellect, and that this association is driven entirely by the Intellect aspect of this factor. Further, we showed through an additive factors design, which partitions stages of processing by systematically manipulating decisional and perceptual qualities of stimuli, that Intellect plays a role in reaction time similar to that of general intelligence. For both cognitive ability and Intellect, higher scores were associated solely with the information processing stage of the production of speeded responses.

However, perhaps the most surprising and unique of our findings is that Intellect, rather than reflecting merely a less accurate self-report of cognitive ability, adds incremental variance above and beyond IQ in predicting objectively measured response times on these tasks. This is consistent with an emergent understanding that Intellect has unique associations with intellectual effort, independently of ability, for example during working memory tasks (Smillie et al. 2016). Although IQ and associated neural processes likely represent one mechanistic component captured by reaction time, the typically reported correlations in the range of −.20 to −.40 (e.g., Jensen 2006) indicate that much of its variance remains unexplained by IQ alone. Intellect, which adds a significant increment of about 3% to IQ in predicting mean RT on the tone task in our sample, may represent one such additional component.

If this finding does reflect a true property of personality variation and is not an artifact that emerges from test properties, it is tempting to speculate as to what underlying individual difference is captured by Intellect that predicts RT above and beyond IQ. Of the 10 items that measure Intellect in the BFAS, four of these retained predictive value on tone RT after controlling for IQ. These four items are:

- I can handle a lot of information. (*p* = .002)
- I like to solve complex problems. (p<.001)
- I think quickly. (p<.001)
- I learn things slowly (reversed). (*p* = .008)

In our view, the most parsimonious interpretation of what these items collectively capture speaks to an oft-overlooked benefit of self report questionnaires. Items such as “I think quickly” may accurately capture an intimate understanding of one’s internal physiology that, despite being subjectively evaluated, are nevertheless psychometrically valid predictors of an objective measurement. Although individuals may be unlikely to assess their own intelligence more reliably than an IQ test, it is certainly plausible that the insight afforded by self-report can better capture more specific elements of processing speed that IQ alone does not.

It is also interesting to note that only one of these four items retained significant predictive value above IQ in the number-comparison task (“I think quickly”, p<.001), in which Intellect correlated similarly with mean RT but mostly vanished when controlling for IQ. While Intellect interacted significantly with the Distance manipulation in the tone task, it failed to do so in the number task; the opposite pattern was found for cognitive ability, with IQ strongly interacting with Distance in the number task but only very weakly in the tone task (Willoughby and Lee 2021). Together, this pattern of results hints at the possibility that Intellect and IQ may reflect variation in different mechanistic components involved in the processing of auditory and numerical stimuli, respectively. Subsequent studies targeted at testing this hypothesis would be better equipped to ascertain its validity.

Although we believe these findings to contribute novel insight to a mechanistic understanding of personality, it is not without its limitations. Our relatively large sample of ∼480 participants nevertheless yielded several correlations of nominal significance that are difficult to interpret on theoretical grounds; a larger sample would be better able to minimize the chance of false discovery in a large suite of multiple comparisons across all Big Five domains and aspects. It also worth considering the possibility that individual differences in effort expenditure may account for these correlations, as effort has been found to be a significant predictor of RT (Stevens et al. 2016).

Our primary hypotheses were targeted at understanding the relation between Openness/Intellect aspects and reaction time, but a more comprehensive test of cognitive ability would likely afford a higher resolution view into its relationship with other variables. For example, our IQ short-form was unable to clarify the differential roles of verbal and nonverbal intelligence in personality, likely because only four items comprised each of these sub-measures. In sum, our findings represent a novel line of support for the cybernetic functions of Intellect and IQ in a comprehensive personality taxonomy, and a potential springboard for psychologists in conducting future research into the role of reaction time in dimensions of personality.

## Figures and Tables

**Table 1:** Descriptive statistics and reliability measures for the Big Five Aspect Scale.

	*M*	*SD*	α	$\omega_{h}$	$\omega_{t}$
Openness/Intellect	3.7	0.47	.76	.70	.83
Openness	3.77	0.62	.83	.72	.87
Intellect	3.64	0.55	.80	.62	.83
Conscientiousness	3.53	0.53	.87	.48	.90
Industriousness	3.32	0.63	.84	.78	.86
Orderliness	3.74	0.62	.83	.57	.87
Extraversion	3.55	0.56	.90	.60	.92
Assertiveness	3.38	0.67	.88	.74	.90
Enthusiasm	3.72	0.64	.86	.66	.89
Agreeableness	4.06	0.44	.87	.71	.89
Politeness	3.89	0.52	.75	.55	.79
Compassion	4.23	0.5	.88	.85	.90
Neuroticism	2.85	0.65	.91	.66	.93
Withdrawal	3.02	0.69	.85	.83	.88
Volatility	2.69	0.76	.90	.75	.92

*Note:* *N* = 477 in current sample with valid data on all tasks. *α* = Cronbach’s alpha, *ω_h_* = omega hierarchical, *ω_t_* = omega total.

**Table 2:** Reliability comparisons of ICAR-16 items in current study and Condon and Revelle, 2014.

	α			$\omega_{h}$			$\omega_{t}$			Items	
	*CR*	*CS*		*CR*	*CS*		*CR*	*CS*		*CR*	*CS*
ICAR-16	.81	.73		.66	.47		.83	.76		16	16
LN	.77	.60		.66	.58		.80	.63		9	4
MR	.68	.46		.58	.44		.71	.57		11	4
R3D	.93	.70		.78	.66		.94	.74		24	4
VR	.76	.40		.64	.39		.77	.44		16	4

*Note:* *CR* = Condon and Revelle (2014), *CS* = Current study; ICAR-16 = Proportion correct for total ICAR sample test, LN = Letter and number sequence items, MR = Matrix reasoning items, R3D = 3D rotation items, VR = Verbal reasoning items; reliability indices are *α* = Cronbach’s alpha, *ω_h_* = omega hierarchical, *ω_t_* = omega total. Values are based on composites of Pearson correlations between items. Total *N* sampled in Condon and Revelle (2014) = 96,958 individuals; *N* = 477 had valid data for all tasks in current sample.

**Table 3:** Distributional characteristics of cognitive assessment and RT moments in current sample.

	*M*	SD	Max	Min	Skew
ICAR-16	.62	.20	1	0	−0.21
*Number task*					
RT *M*	534.2	77.95	386.8	876.2	1.17
RT *SD*	106.5	43.4	42.5	359.5	1.86
Accuracy	.981	.015	.928	1.00	−0.91
*Tone task*					
RT *M*	609.6	184.1	342.8	1908.2	2.58
RT *SD*	172.5	130.3	45.0	1180.5	3.55
Accuracy	.939	.057	.466	1.00	−3.46

**Table 4:** Correlations between measures of cognitive ability and the Openness/Intellect domain and aspects in current sample.

		Verbal	Matrix	Letters	3D
	*g*	Reasoning	Reasoning	& Numbers	Rotation
Openness/Intellect	.14 ***	.09	.13 ***	.08	.09
Intellect	.24 ***	.22 ***	.15 ***	.14 ***	.16 ***
Openness	−.01	−.07	.06	−.01	−.01

*Note:* *N* = 477 had valid means data for all tasks in current sample. “*g*” refers to total proportion correct over all 16 items. *** denotes *p* < .005.

**Table 5:** Correlation matrix showing all interrelationships between Big Five personality factors and their respective aspects, ICAR-16 cognitive ability score, and reaction time moments (mean, *SD*, and accuracy) for number and tone comparison tasks.

	O				C				E				A				N				ICAR-16		Number RT				Tone RT		
	1	1a	1b		2	2a	2b		3	3a	3b		4	4a	4b		5	5a	5b		6		7a	7b	7c		8a	8b	8c
1. Openness/Intellect																													
1a. Intellect	.76 ***																												
1b. Openness	.82 ***	.25 ***																											
2. Conscientiousness	−.04	.16 ***	−.21 ***																										
2a. Industriousness	.04	.31 ***	−.21 ***		.85 ***																								
2b. Orderliness	−.12 *	−.04	−.14 ***		.85 ***	.45 ***																							
3. Extraversion	.24 ***	.27 ***	.12 **		.24 ***	.31 ***	.08																						
3a. Enthusiasm	.12 **	.13 ***	.07		.15 ***	.24 ***	.02		.86 ***																				
3b. Assertiveness	.29 ***	.33 ***	.14 ***		.25 ***	.30 ***	.13 **		.87 ***	.49 ***																			
4. Agreeableness	.22 ***	.09	.25 ***		.08	.07	.08		.15 ***	.36 ***	−.09																		
4a. Compassion	.35 ***	.19 ***	.35 ***		.05	.04	.04		.34 ***	.46 ***	.13 ***		.85 ***																
4b. Politeness	.03	−.04	.08		.09 *	.07	.09		−.07	.16 ***	−.28 ***		.86 ***	.46 ***															
5. Neuroticism	−.06	−.24 ***	.12 **		−.26 ***	−.45 ***	.02		−.29 ***	−.29 ***	−.21 ***		−.11 *	−.03	−.16 ***														
5a. Withdrawal	−.02	−.22 ***	.16 ***		−.32 ***	−.51 ***	−.02		−.44 ***	−.36 ***	−.39 ***		.07	.06	.06		.88 ***												
5b. Volatility	−.08	−.21 ***	.06		−.15 ***	−.30 ***	.04		−.10 *	−.17 ***	−.01		−.25 ***	−.10 *	−.33 ***		.90 ***	.58 ***											
6. ICAR-16	.14 ***	.24 ***	−.01		.02	.00	.04		−.08	−.11 *	−.03		−.06	−.03	−.07		−.05	−.06	−.03										
*Number task*																													
7a. RT M	−.13 ***	−.18 ***	−.04		−.07	−.08	−.04		−.05	−.02	−.06		.01	−.02	.04		.04	.07	.01		−.38 ***								
7b. RT SD	−.11 *	−.16 ***	−.03		−.08	−.09	−.05		−.03	.00	−.05		.03	.00	.04		.03	.06	.01		−.29 ***		.88 ***						
7c. Accuracy	−.04	−.07	.01		.12 **	.08	.13 ***		.06	.09 *	.01		.13 ***	.12 *	.10 *		.04	.04	.03		−.03		.17 ***	.02					
*Tone task*																													
8a. RT M	−.16 ***	−.21 ***	−.05		−.01	−.04	.03		−.03	−.02	−.03		.00	−.02	.02		.03	.07	−.01		−.27 ***		.60 ***	.53 ***	.24 ***				
8b. RT SD	−.15 ***	−.19 ***	−.05		−.02	−.08	.04		−.04	−.03	−.05		.01	−.02	.04		.03	.09	−.02		−.23 ***		.51 ***	.52 ***	.13 ***		.92 ***		
8c. Accuracy	.13 ***	.16 ***	.05		.07	.06	.07		.09 *	.11 *	.05		.12 **	.13 ***	.08		−.01	−.02	.01		.27 ***		−.24 ***	−.18 ***	.06		−.25 ***	−.24 ***	

*Note:* * denotes *p* < .05, ** *p* < .01, and *** *p* < .005.

**Table 6:** Comparison of correlations with Openness versus Intellect using confidence intervals.

			Number Task				Tone Task		
	*g*		RT *M*	RT *SD*	Acc		RT *M*	RT *SD*	Acc
95% CI	[.14, .36]		[−.25, −.03]	[−.24, −.02]	[−.19, .03]		[−.27, −.06]	[−.26, −.04]	[.00, .22]
99.5% CI	[.10, .40]		[−.30, .01]	[−.28, .03]	[−.23, .08]		[−.32, −.01]	[−.30, .01]	[−.05, .26]

*Note:* Confidence intervals were calculated using Zou (2007)’s method, which provides a comparison of two overlapping correlations based on dependent groups. Openness aspect correlations are subtracted from those of the Intellect aspect.

**Table 7:** Means and standard deviations (in parentheses) for reaction time (ms) and accuracy (proportion correct) across both manipulations’ levels of difficulty for number and tone comparison tasks.

	Number Task			Tone Task	
Manipulation	*M* RT (*SD*)	Acc (*SD*)		*M* RT (*SD*)	Acc (*SD*)
*Contrast*				
Hardest	563 (96)	.98 (.05)		622 (207)	.93 (.12)
Hard	535 (93)	.98 (.05)		605 (217)	.94 (.11)
Easy	520 (92)	.98 (.05)		602 (226)	.95 (.10)
Easiest	519 (92)	.98 (.05)		605 (216)	.94 (.11)
*Distance*				
Hardest	575 (104)	.96 (.07)		695 (269)	.85 (.15)
Hard	544 (93)	.98 (.05)		622 (213)	.94 (.09)
Easy	513 (84)	.99 (.03)		573 (183)	.98 (.06)
Easiest	504 (78)	.99 (.02)		545 (152)	.99 (.05)

*Note:* “Distance” refers to numerical and frequency distance from target stimulus and “Contrast” refers to visual contrast of numeral and loudness of tone in number and tone tasks, respectively. *N* = 476 had valid data for all combinations of conditions.

**Figure A2:**
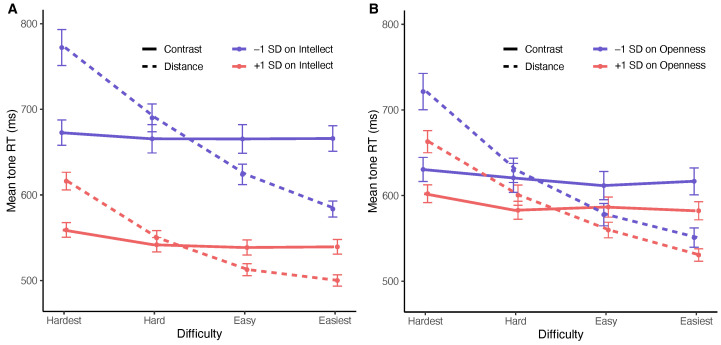
Mean tone-task RT for each condition for participants 1 SD and above and −1 SD and below the group mean for Intellect (**A**) and Openness (**B**). Aspect scores represent standard scores. Error bars represent ±1 standard error of the sample mean.

## Data Availability

This project was preregistered on the Open Science Framework (https://osf.io/q792f/?view_only=33e1fb84883045bb9be6a4d2dff35e55; accessed 18 February 2021). Additionally, open anonymized data and *R* code used in key analyses are available on the Open Science Framework here: https://osf.io/eys87/?view_only=f89ea980361f4863a29dd20dcf32d763 (accessed 20 May 2022).

## Appendix A. Methodological Details

### Appendix A.1. Sample

Participants were recruited through the undergraduate psychology recruitment pool at the University of Minnesota. A total of 481 participants took part in the experiment over a 3-year period; of these, 477 had data for all four tasks (77.78% female, 20.75% male, 1.47% missing/other).

To account for well known ontogenic changes in reaction time (Thompson et al. 2014), individuals were recruited from the 18–24 age range (M=19.6, SD=1.6). A total of six individuals participated whose ages fell within the 25–30 range. We conducted *t*-tests to evaluate whether these individuals significantly differed from their younger cohort on RT moments; no significant differences were found, so these individuals were not excluded from analysis. For the number task, the difference in mean RT was *t*(475) = –0.13 (*p* = .90), the difference in SD *t*(475) = 0.49 (*p* = .64), and the difference in accuracy *t*(475) = 1.9 (*p* = .11). For the tone task, these differences were *t*(475) = 0.31 (*p* = .77), *t*(475) = 0.05 (*p* = .96), and *t*(475) = 0.35 (*p* = .74), respectively.

### Appendix A.2. Data Filtering

We aimed to filter our data as little as possible in order to capture the full range of variability in response times and accuracy across our participants. Nevertheless, some minor filtering criteria were applied to ensure the reliability and validity of the data. Although it is standard to exclude very fast RTs between 100 ms and 200 ms (Whelan 2008) for reaction time data as these likely do not reflect the process of interest, Luce (1986) argued that valid RTs have a minimum value of at least 100 ms, which can be considered the bare minimum of time needed for physiological processes such as stimulus perception and for motor responses. We have therefore excluded trials faster than 100 ms.

The best method of trimming slow responses has been a subject of debate in reaction time research. Ratcliff (1993) argued that eliminating RTs above an absolute point of cutoff would reduce power in cases where individual response variability was relevant to the effect of interest; in such cases, eliminating slow outliers based on each individual’s distribution would better preserve real data. Given our lack of a priori assumptions of the effects of variability on our analyses of interest, we opted for a conservative method of eliminating individual trial outliers that fall above five standard deviations above that individual’s mean. No data transformations were applied to our RT data.

**Number comparison task.** The number comparison task yielded a total of 71,550 individual trials across 477 participants prior to filtering. Only one individual trial was excluded for being below 100 ms. A total of 1378 incorrect trials were excluded from analysis. Slow outliers were trimmed at 5 standard deviations above each individual’s reaction time mean, which excluded an additional 269 individual trials. These filtering criteria led to a total of 69,902 valid and correct trials across 477 participants.

**Tone comparison task.** For this task, 482 participants generated a total of 72,300 trials prior to filtering. A total of 63 individual trials below 100 ms and a total of 4550 incorrect trials were excluded from further analyses. Slow outliers were trimmed at 5 standard deviations above each individual’s reaction time mean, which excluded an additional 408 individual trials. Additionally, one participant was excluded from data analysis for having too few correct RT trials per condition to participate in ANCOVA and other analyses. These criteria netted a final sample of 67,227 valid and correct tone task trials across 481 individuals.

**ICAR-16 sample test.** 483 participants took the short-form cognitive assessment. No participants were excluded based on unusual scores; for example, the one person who scored 0 out of 16 possible items was included because the time taken to complete the test was not unusually fast for this individual and a score of 0 was well within the normal distributional range of scores. No participants yielded incomplete data on the 16 items.

A total of 477 participants had valid data across all conditions for the BFAS, the ICAR-16 sample test, the number comparison task, and the tone comparison task.

### Appendix A.3. Design and Procedure

Experimenters were instructed to monitor a variety of potential sources of error. They verbally confirmed with all participants their age, the status of their vision as normal or normal-corrected, and their status as native English speakers. Participants were given both printed and verbal instructions for the tasks, and were required to verbally affirm that they had no questions or uncertainties about the task procedure before they began. Participants were also instructed to administer keypresses with their dominant hand, to use only the pointer and middle fingers, and to keep fingers on the keys at all times. Participants then answered a short computerized questionnaire to confirm their age and sex before beginning the experiment.

Our goal in this study was to employ additive-factors logic to compare the effects of two manipulations on task performance. Both manipulations were intended to slow down performance, but only one was intended to achieve this slowdown by varying the difficulty of discriminating the trial stimulus from other members of the stimulus set. The second manipulation, by contrast, varied the difficulty of perceiving the stimulus.

Our number-comparison task is adapted from Moyer and Landauer (1967). This task requires that participants respond as quickly and accurately as possible to whether a stimulus digit is less than or greater than an invariant target digit, which was 5 in our implementation for Experiment 1. Participants were instructed to press the *Q* key on the keyboard if the number that appeared onscreen was less than 5, and the *W* key if the number is greater than 5. A keypress terminated the trial, prompted brief feedback (correct or incorrect), and initiated the next trial.

The stimulus digits (1 through 9 excluding 5) were randomized across trials. Additionally, the four levels of Contrast were also randomized across trials to produce a total of 16 Distance × Contrast combinations. Participants first completed 30 practice trials, then 3 blocks of 50 trials, each of which were separated by short breaks. A given combination of digit and level of Contrast could only appear a maximum of three trials in a row in order to mitigate the unwanted influence of stimulus repetition (e.g., Kraut and Smothergill 1978).

The tone comparison task is designed to be structured to the number-comparison task. Following a randomized foreperiod delay between 1200 and 1900 ms, two tones are presented sequentially to the participant through the set of headphones connected to the computer. The first tone is the target, which is positioned at the center of the distribution of stimulus frequencies (660 Hz) and at normal speaking volume (50 dB). The second tone is the stimulus, which can be any combination of frequency and loudness from the set of 16 options, excepting the target frequency of 660 Hz. The participant is instructed to respond as quickly and accurately as possible by pressing the “Q” key on the keyboard if the second tone is higher in pitch than the target tone, and the “W” key if the stimulus tone is lower in pitch. A keypress terminates the trial and initiates brief feedback (correct or incorrect) before continuing to the next trial.

Eight total stimulus frequencies were used, four above and four below the target frequency; in this condition “distance” is coded as distance from the target frequency for four levels of “distance” and a total of 16 different conditions per participant. The four levels of both loudness and frequency distance are randomized and counterbalanced across trials. Each stimulus frequency and loudness level can appear a maximum of three trials in a row. Participants first complete 30 practice trials, then 3 blocks of 50 trials each which are separated by short breaks.

### Appendix A.4. Apparatus and Stimuli

Our goal in this study was to employ additive-factors logic to compare the effects of two manipulations on task performance. Both manipulations were intended to slow down performance, but only one was intended to achieve this slowdown by varying the difficulty of discriminating the trial stimulus from other members of the stimulus set. The second manipulation, by contrast, varied the difficulty of perceiving the stimulus.

The first experimental paradigm focuses on visual processing of numerals displayed on a computer screen. We chose a number-comparison task from the field of numerical cognition, where detailed mechanistic models of robust phenomena have already been proposed (Cohen Kadosh and Dowler 2015; Dehaene 2007). In such a task, participants are given a target digit that is invariant across all trials. In any particular trial, they must respond via keypress whether the stimulus digit is less than or greater than the digit.

For the perceptual manipulation, we varied the contrast of the numerals against the background across four levels; lower contrast produces slower responses. This manipulation was intended to target an early stage of visual processing that should occur temporally prior to any processing of informational content. That this manipulation would target an early stage of visual processing is supported by many lines of converging evidence (Campbell et al. 1973; De Valois et al. 1982; Hubel and Wiesel 1968; Sigman and Dehaene 2005).

**Stimulus presentation.** Stimuli were administered through the E-Prime 2.0 software (Schneider et al. 2001) running on one of three identical Apple iMac computers with identical hardware. Visual stimuli were displayed on a 24-inch Apple monitor (60 Hz refresh rate; 1920 × 1080 pixel resolution), and participants’ responses were made on a computer keyboard.

For Experiment 1, the stimulus of interest was a single-digit number between 1 and 9 (excluding 5), which appeared at the center of the computer screen following a fixation cross and a randomized foreperiod delay between 1200 and 1900 milliseconds. Each displayed digit additionally varied in its visual contrast against an off-white background on the computer screen. Four levels of contrast were used, ranging from lightest grey (hexidecimal 215,215,215), light grey (hexidecimal 210,210,210), dark grey (hexidecimal 170,170,170), to darkest grey (hexidecimal 165,165,165). All digits were presented in 72-point Arial font on an off-white background screen (hexidecimal 220,220,220) in E-Prime 2.0.

## Appendix B. Extended Results

### Appendix B.1. Validity Analyses

**ICAR-16: Confirmatory factor analysis.** We used confirmatory factor analysis to test a four-factor model of the ICAR-16 composed of letter and number sequences, matrix reasoning, 3D rotation and verbal reasoning. This model was fit using the lavaan package in *R* (Rosseel 2012) with full-information maximum likelihood (FIML). Model fit was strong, with a Tucker-Lewis Index (TLI) of .978, a Root Mean Square Error of Approximation (RMSEA) of .017 (90% CI: .000, .031), and a Standardized Root Mean Square Residual (SRMR) of .033. The full four-factor model fit the data significantly better than a single-factor solution ($\chi_{6}^{2}$ difference between models = 181.71; AIC difference = 172.2; BIC difference = 147.2; p<.001), and better than a four-factor solution that did not allow covariances among the four latent factors ($\chi_{6}^{2}$ difference between models = 184.21, AIC difference = 169.7; BIC difference = 144.7; p<.001). As expected, the indicators all showed significant positive factor loadings, with standardized coefficients ranging from .299 to .663 (Table A1).

To account for the possibility that ordinal response data biases the results of a continuous estimator, we also applied the weighted least square mean and variance adjusted (WLSMV) estimator for ordinal data (Li 2016). This method yields superior fit to FIML. For the four-factor model, the WLSMV estimator produced a TLI of .997, an SRMR of .059, and an RMSEA of .008 (90% CI: 0, .026), which was in turn superior to the WLSMV fit for the one-factor model (TLI = .725; SRMR = .057; RMSEA = .062 [90% CI .054, .070]; $\chi_{6}^{2}$ difference between models = 96.307, p< .001).

**Table A1:** Descriptive statistics and standardized factor loadings (β) of each ICAR-16 item on its own latent factor in the current sample. All loadings are significant at p<.001.

Latent Factor	Indicator	*M*	SD	β
Letter & Number	LN.7	.79	.40	.43
Letter & Number	LN.33	.75	.43	.60
Letter & Number	LN.34	.78	.41	.57
Letter & Number	LN.58	.56	.50	.49
Matrix Reasoning	MR.45	.66	.47	.47
Matrix Reasoning	MR.46	.72	.45	.49
Matrix Reasoning	MR.47	.79	.41	.33
Matrix Reasoning	MR.55	.39	.49	.42
3D Rotation	R3D.3	.28	.45	.61
3D Rotation	R3D.4	.40	.49	.66
3D Rotation	R3D.6	.46	.50	.56
3D Rotation	R3D.8	.28	.45	.58
Verbal Reasoning	VR.4	.84	.37	.49
Verbal Reasoning	VR.16	.63	.48	.30
Verbal Reasoning	VR.17	.84	.37	.37
Verbal Reasoning	VR.19	.71	.45	.39

*Note:* R3D = Three-dimensional Rotation, LN = Letter And Number series, VR = Verbal Reasoning, MR = Matrix Reasoning.

Additionally, we observed significant positive correlations among all four latent factors (Table A2), indicating that participants who showed high ability in one dimension were more likely to show high ability in the others as well. Taken together, these results are consistent with use of the ICAR-16 as a good short-form measure of cognitive ability, with the advantage of its short administration time outweighing its limitations in the context of this study.

**Big Five Aspect Scale: Confirmatory factor analysis.** We used confirmatory factor analysis to test both a 5-factor (Big Five) model and a 10-factor (Aspects) model of response data from the Big Five Aspect Scale. These models were fit using the lavaan package in *R* with full-information maximum likelihood (FIML) and the weighted least square mean and variance adjusted (WLSMV) estimator for ordinal data (Li 2016). Likely due to the BFAS’s close approximation of continuous data (Figure A1), relative model fit was similar for both methods. For FIML, model fit for the 5-factor solution gave a TLI of .544 and RMSEA of .062 (90% CI: .061, .064), and a TLI of .710 and RMSEA of .050 (90% CI: .049, .051) for a 10-factor aspect model. The WLSMV estimator produced a TLI of .649 and RMSEA of .060 (90% CI: .059, .062) for the five-factor model, and a TLI of .802 and RMSEA of .045 (90% CI: .044, .046) for the 10-aspect model. The 10-aspect model fit the data significantly better than the traditional five-factor solution for both the FIML estimator ($\chi_{35}^{2}$ difference between models = 3411.4, p<.001) and the WLSMV estimator ($\chi_{35}^{2}$ difference between models = 1280.8, p<.001).

**Table A2:** Latent factor correlations for the ICAR-16 in the current sample.

Factor 1	Factor 2	Correlation	*p*-Value
LN	MR	.555	<.001
LN	R3D	.417	<.001
LN	VR	.580	<.001
MR	R3D	.423	<.001
MR	VR	.624	<.001
R3D	VR	.626	<.001

*Note:* Primary factor loadings for each item are shown in bold. R3D = Three-dimensional Rotation, LN = Letter And Number series, VR = Verbal Reasoning, MR = Matrix Reasoning.

#### Interactions with Aspects

To further examine the relationship between reaction time at varying levels of Contrast and Distance difficulty with the Openness and Intellect aspects (Figure 1), we examined mean tone-task RTs for each condition in the top ±1 SD above and below the sample mean in Intellect or Openness. Figure A2 shows mean reaction time in the tone task for each level of both Distance and Contrast factors for participants ±1 SD and above/below the sample mean for Intellect (A) and Openness (B). Note that while especially high and low scores on Intellect pull apart the reaction time slopes, this is far less the case for high and low scores on Openness.

**Table A3:** Matrix of Bonferroni-corrected *p*-values from pairwise correlations shown in Table 5.

	O				C				E				A				N				ICAR-16		Number RT				Tone RT		
	1	1a	1b		2	2a	2b		3	3a	3b		4	4a	4b		5	5a	5b		6		7a	7b	7c		8a	8b	8c
1. Openness/Intellect																													
1a. Intellect	< .001																												
1b. Openness	< .001	< .001																											
2. Conscientiousness	–	.173	.003																										
2a. Industriousness	–	< .001	.002		< .001																								
2b. Orderliness	–	–	–		< .001	< .001																							
3. Extraversion	< .001	< .001	–		< .001	< .001	–																						
3a. Enthusiasm	–	–	–		.358	< .001	–		< .001																				
3b. Assertiveness	< .001	< .001	–		< .001	< .001	–		< .001	< .001																			
4. Agreeableness	.001	–	< .001		–	–	–		.438	< .001	–																		
4a. Compassion	< .001	.011	< .001		–	–	–		< .001	< .001	–		< .001																
4b. Politeness	–	–	–		–	–	–		–	.232	< .001		< .001	< .001															
5. Neuroticism	–	< .001	–		< .001	< .001	–		< .001	< .001	.001		–	–	.195														
5a. Withdrawal	–	.001	.263		< .001	< .001	–		< .001	< .001	< .001		–	–	–		< .001												
5b. Volatility	–	.003	–		.413	< .001	–		–	.090	–		< .001	–	< .001		< .001	< .001											
6. ICAR-16	–	< .001	–		–	–	–		–	–	–		–	–	–		–	–	–										
*Number task*																													
7a. RT M	–	.031	–		–	–	–		–	–	–		–	–	–		–	–	–		< .001								
7b. RT SD	–	.262	–		–	–	–		–	–	–		–	–	–		–	–	–		< .001		< .001						
7c. Accuracy	–	–	–		–	–	–		–	–	–		–	–	–		–	–	–		–		.060	–					
*Tone task*																													
8a. RT M	.304	.002	–		–	–	–		–	–	–		–	–	–		–	–	–		< .001		< .001	< .001	< .001				
8b. RT SD	.614	.009	–		–	–	–		–	–	–		–	–	–		–	–	–		< .001		< .001	< .001	–		< .001		
8c. Accuracy	–	.221	–		–	–	–		–	–	–		–	–	–		–	–	–		< .001		< .001	.053	–		< .001	< .001	

*Note:* Entries showing “–” indicate *p*-values indistinguishable from 1.

### Appendix B.2. Bonferroni Corrections

To account for multiple comparisons, *p*-values indicated from correlations in Table 5 were adjusted using a Bonferroni correction. These results are shown in Appendix B Table A3.
